# Safety and Efficacy of Tirofiban During Intravenous Thrombolysis Bridging to Mechanical Thrombectomy for Acute Ischemic Stroke Patients: A Meta-Analysis

**DOI:** 10.3389/fneur.2022.851910

**Published:** 2022-04-29

**Authors:** Wei Li, Guohui Lin, Zaixing Xiao, Yichuan Zhang, Bin Li, Yu Zhou, Erqing Chai

**Affiliations:** ^1^The First Clinical Medical College of Gansu University of Chinese Medicine, Lanzhou, China; ^2^Cerebrovascular Disease Center of Gansu Provincial People's Hospital, Lanzhou, China; ^3^Key Laboratory of Cerebrovascular Diseases in Gansu Province, Lanzhou, China; ^4^Day Treatment Center II of Gansu Provincial Maternity and Child-Care Hospital, Lanzhou, China; ^5^The First School of Clinical Medicine of Lanzhou University, Lanzhou, China

**Keywords:** tirofiban, acute ischemic stroke, intravenous thrombolysis, mechanical thrombectomy, meta analysis

## Abstract

**Introduction:**

The safety and efficacy of tirofiban in intravenous thrombolysis (IVT) bridging to mechanical thrombectomy in patients with acute ischemic stroke (AIS) is unknown. The purpose of this meta-analysis was to evaluate the safety and efficacy of tirofiban in IVT bridging to mechanical thrombectomy in acute ischemic stroke.

**Methods:**

We systematically searched PubMed, EMBASE, Web of Science, and The Cochrane Library, CNKI, and Wan Fang databases for randomized controlled trials and observational studies (case-control studies and cohort studies) comparing the tirofiban and non-tirofiban groups in AIS intravenous thrombolysis bridging to mechanical thrombectomy (Published by November 20, 2021). Our primary safety endpoints were symptomatic cerebral hemorrhage (sICH), intracranial hemorrhage (ICH), postoperative re-occlusion, and 3-month mortality; the efficacy endpoints were 3-month favorable functional outcome (MRS ≤ 2) and successful recanalization rate (modified thrombolytic therapy in cerebral infarction (mTICI) 2b or 3).

**Results:**

A total of 7 studies with 1,176 patients were included in this meta-analysis. A comprehensive analysis of the included literature showed that the difference between the tirofiban and non-tirofiban groups in terms of successful recanalization (OR = 1.19, 95% Cl [0.69, 2.03], *p* = 0.53, *I*^2^ = 22%) and favorable functional outcome at 3 months (OR = 1.13, 95% Cl [0.81, 1.60], *p* = 0.47, *I*^2^ = 17%) in patients with IVT bridging mechanical thrombectomy of AIS was not statistically significant. Also, the differences in the incidence of sICH (OR = 0.97, 95% Cl [0.58, 1.62], *p* = 0.89) and ICH (OR = 0.83, 95% Cl [0.55, 1.24], *p* = 0.36) between the two groups were not statistically significant. However, the use of tirofiban during IVT bridging mechanical thrombectomy reduced the rate of postoperative re-occlusion (OR = 0.36, 95% Cl [0.14, 0.91], *p* = 0.03) and mortality within 3 months (OR = 0.54, 95% Cl [0.33, 0.87], *p* = 0.01) in patients.

**Conclusion:**

The use of tirofiban during IVT bridging mechanical thrombectomy for AIS does not increase the risk of sICH and ICH in patients and reduces the risk of postoperative re-occlusion and mortality in patients within 3 months. However, this result needs to be further confirmed by additional large-sample, multicenter, prospective randomized controlled trials.

**Systematic Review Registration:**

http://www.crd.york.ac.uk/PROSPERO/, identifier: CRD42022297441.

## Introduction

Acute ischemic stroke (AIS) is one of the major diseases threatening human health, characterized by high morbidity, disability and mortality. Early recovery of cerebral blood flow perfusion and rescue of ischemic penumbra are the only effective treatment methods. Intravenous thrombolysis (IVT) is used as the preferred modality to open blood vessels in the hyper acute phase (3–4.5 h) of ischemic stroke (1), but it's application is limited by the time window and complex contraindications.

In recent years, with the rapid development of endovascular therapy (EVT) techniques to improve cerebrovascular revascularization rates and expand the therapeutic window, five studies (2–6) with data from randomized trials have shown that mechanical thrombectomy (MT) can be the standard of care for large vessel occlusion (LAO) opening, with revascularization rates ranging from 58.7 to 88%. Although MT has a high success rate in revascularization of patients with large vessel occlusion, improving functional outcome and reducing mortality, occlusion still occurs early after recanalization in ~20% of patients (7), most of whom have a combination of severe atherosclerotic stenosis or endothelial injury causing platelet aggregation leading to thrombotic events and early re-occlusion (8, 9). GPIIb/ IIIa receptor antagonist tirofiban has been widely investigated by a wide range of investigators for its effectiveness in blocking the final pathway of platelet aggregation and thrombosis (10), aiming to prevent arterial re-occlusion and thromboembolic complications after early MT surgery.

The efficacy of tirofiban in inhibiting platelet aggregation has been well established in acute coronary syndromes (11), but there are no consistent findings in clinical studies on the safety and efficacy of intra-cerebrovascular therapy (12, 13), with major controversy over whether the benefit in clinical outcomes of tirofiban use during surgery in patients with IVT bridging to mechanical thrombectomy outweighs the self-induced bleeding wind limit. Moreover, the American Heart Association/American Stroke Association (AHA/ASA) guidelines do not recommend the use of antiplatelet agents within 24 h after IVT because of concerns about increased bleeding complications (14). In this study, this meta-analysis of randomized trials and observational studies (case-control studies and cohort studies) of tirofiban for IVT bridging mechanical thrombectomy in acute ischemic stroke was conducted to assess the safety and efficacy of it.

## Methods

### Search Strategy

This meta-analysis was performed according to the PRISMA guidelines. We systematically searched PubMed, EMBASE, Web of Science, The Cochrane Library, CNKI, and WanFang databases for randomized controlled trials and observational studies (case-control studies and cohort studies) comparing the use of the tirofiban group with the non-tirofiban group (blank group) in the AIS intravenous thrombolysis bridging MT (Published by November 20, 2021). Two investigators independently conducted a literature search and we used a combination of the following terms: Ischemic Stroke (Mesh), Ischemic Strokes, Stroke, Ischemic, Ischemic Stroke, Wake-up Stroke, Acute Ischemic Stroke, Acute Ischemic Strokes, large vessel occlusion, large artery occlusions, Tirofiban(Mesh), Aggrastat, MK-383, L-700462, Hydrochloride, Tirofiban Hydrochloride Monohydrate, GP IIb/IIIa receptor antagonist, mechanical thrombectomy, intravenous thrombolysis, bridging therapy, Safety, efficacy, randomized controlled trial, observational studies. References generated from these searches were imported into the reference manager EndNote X9.3.1 (Thompson Reuters, Philadelphia, PA), and duplicate references were removed. Then, journal article titles and abstracts were systematically screened by 2 researchers independently according to the following inclusion and exclusion criteria. This meta-analysis has been registered in PROSPERO (ID: CRD42022297441).

### Inclusion Criteria

(1) Patients with confirmed AIS; (2) patients treated with IVT within the time window (3–4.5 h after stroke); (3)Data are clearly available in the literature for two treatment groups: (i) IVT bridging to MT + tirofiban and (ii) IVT bridging to MT only; (4) MT included contact aspiration, stent retriever, permanent intracranial stenting, and balloon angioplasty; (5) The modes of administration of tirofiban include: arterial administration alone, intravenous administration alone, combined arterial and venous administration; (6) Randomized controlled trials and observational studies (case-control studies and cohort studies).

### Exclusion Criteria

(1) Unpublished studies, conference abstracts, letters, reviews, correspondence, and animal studies; (2) studies with duplicate or overlapping data; (3) lack of outcome data other than hospitalization; (4) Literatures that do not provide data for both treatment groups: (i) IVT bridging to MT + tirofiban and (ii) IVT bridging to MT only (5) all case series of <10 patients.

### Tirofiban Administration During Mechanical Thrombectomy

In the perioperative period for patients undergoing mechanical Thrombectomy, tirofiban is generally used in the following situations. (1) Emergency stent placement for severe residual stenosis or immediate re-occlusion; (2) Balloon angioplasty for severe residual stenosis or immediate re-occlusion; (3) successful recanalization with ≥ 3 passes with stent retriever for presumed endothelial damage or instant reocclusion; (4) Severe *in situ* atherosclerosis with high risk of early re-occlusion. Unless cerebral hemorrhage is suspected, the standard procedure is a small intra-arterial dose followed by a continuous intravenous infusion for 24 h, with the exact dose administered at the discretion of the interventionist.

### Data Extraction and Efficacy Metrics

Data for each eligible literature were extracted independently by 2 investigators, and any disagreements were resolved by discussion and consultation with a 3rd senior neurosurgeon. Basic characteristics such as first author's name, study design, sample size, mean age, sex ratio, intravenous thrombolysis, MT method, the reasons for administration of tirofiban, and tirofiban dosing strategy were extracted using a pre-developed form. The primary efficacy analyzed was the 3-month favorable functional outcome, defined as MRS ≤ 2, and the secondary efficacy outcome was the successful recanalization rate [modified thrombolytic therapy in cerebral infarction (mTICI) 2b or 3]. Safety outcomes mortality at 3 months, postoperative re-occlusion, intracranial hemorrhage, and symptomatic cerebral hemorrhage (sICH), which was defined as cerebral hemorrhage associated with clinical deterioration (≥4-point increase in NIHSS score) according to ECASS-III (15).

### Literature Quality Assessment

Each of the two trained researchers read all the titles and abstracts of the literature, first screened out the literature that clearly did not meet the inclusion criteria, and then read the full text of the literature to initially identify the literature that could be included in the study. Finally, the screening results of the two researchers were cross-checked, and the two evaluators discussed the questionable literature and combined the third-party opinions to decide whether to include it or not. The quality of randomized controlled trials was evaluated using Risk-of-bias tool (RoB 2.0), and the quality of observational studies was evaluated using the Newcastle-Ottawa Scale.

### Statistics Analysis

Statistical analyses were performed using Review Manager (v.5.3), and differences were considered statistically significant at *P* ≤ 0. 05 if not explicitly stated. We calculated the odds ratio (OR) for categorical variables using a random-effects model, and heterogeneity was evaluated using chi-square tests and *I*^2^ tests, when *I*^2^ < 30% was defined as low heterogeneity; otherwise, it was medium to high heterogeneity. Sensitivity analysis was performed by omitting studies one by one to assess the effect of each study on the overall results. Symmetry was assessed using Begg's and Egger's tests, and significant publication bias was defined as *p* < 0.1, and publication bias was assessed with sensitivity analysis using STATA (v.12).

## Results

### Search Results and Selection of Research Subjects

**Figure 1:**
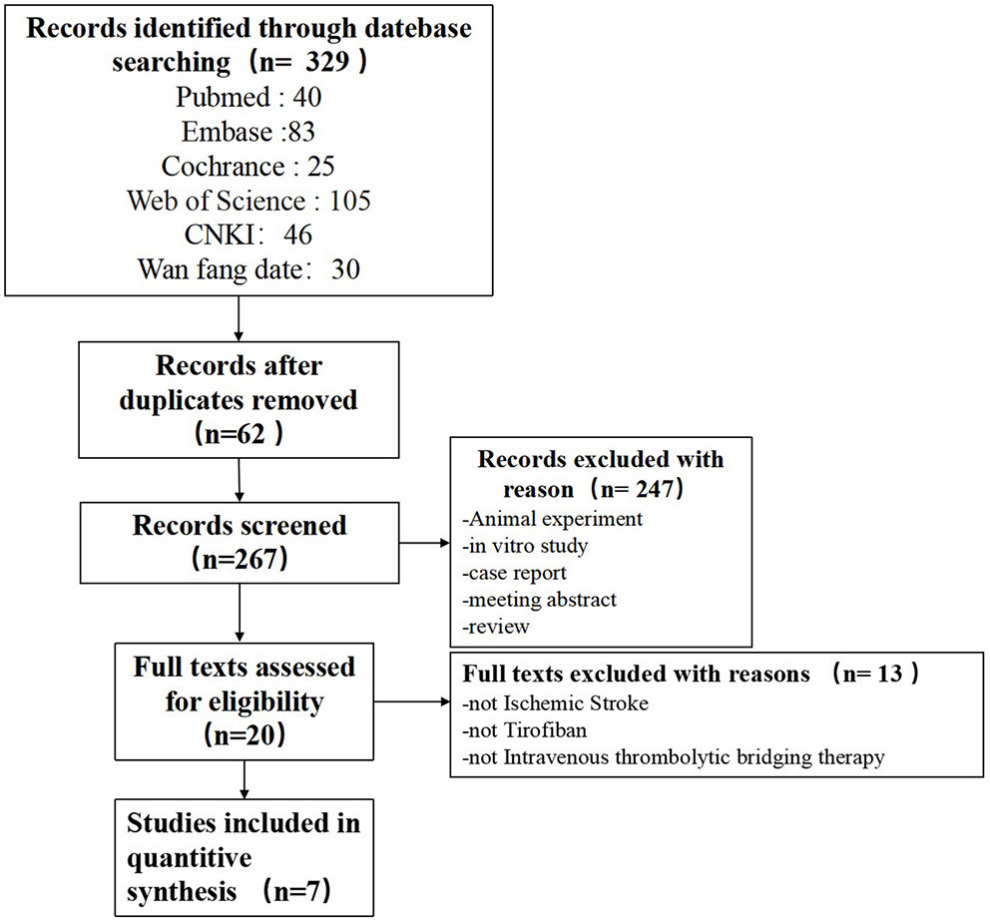
Flow chart of the search and inclusion of literature.

A search from databases identified 329 papers (Pubmed:40, EMBASE: 83, The Cochrane Library: 25, Web of Science: 105, CNKI 46, Wanfang: 30), of which 62 duplicates were excluded, the titles and abstracts of the shortlisted papers were reviewed and another 247 papers, and the remaining 20 papers were read in full-text detail to determine whether they met the inclusion/exclusion criteria, and ultimately, 7 eligible (16–22) papers were included in this meta-analysis (shown in Figure 1).

### Basic Characteristics of the Research Object

One thousand one hundred and seventy six patients from seven (16–22) studies [one (22) randomized controlled trial and six (16–21) observational studies] were included in the final analysis, of which 340 patients (28.9%) were treated with tirofiban. Basic characteristics of the included studies are shown in Table 1.

**Table 1:** Basic characteristics of the included studies.

	**Study design**	**Sample size**		**Mean age, years (T/N)**	**Gender(M/F)**		**Intravenous thrombolysis**	**Endovascular therapy**	**The reasons for administration of tirofiban**	**Tirofiban protocol**
		**T**	**N**		**T**	**N**				
Yi et al. (16)	Observational	22	111	na	na		IV t-PA	Stent retrieval	According to “tirofiban administration during mechanical thrombectomy”	A intra-arterial bolus (0.25–1 mg) followed by continuous intravenous infusion of 0.05 μg/kg/min for 24 h
Huo et al. (17)	Observational	55	152	62.5/64.4	38/17	104/48	IVT	Stent retrieval or contact aspiration	According to “tirofiban administration during mechanical thrombectomy”	A intra-arterial bolus (0.25–1 mg) followed by continuous intravenous infusion of 0.1 μg/kg/min for 12–24 h
Jang et al. (18)	Observational	35	279	66/69	24/11	144/135	IV t-PA	Stent retrieval or contact aspiration	According to “tirofiban administration during mechanical thrombectomy”	A bolus at artery of 1 mL/min (dose range of 0.25–2.0 mg)
Ma et al. (19)	Observational	81	120	62/65	52/29	78/42	IVT	Stent retrieval or contact aspiration	According to “tirofiban administration during mechanical thrombectomy”	A intra-arterial bolus (0.25–1 mg) followed by continuous intravenous infusion of 0.1 μg/kg/min for 24 h
Gao et al. (20)	Observational	45	76	Na	na		IV t-PA	Stent retrieval	Determined by study protocol	A intra-arterial bolus (6 ug/kg) followed by continuous intravenous infusion of 0.1 μg/kg/min for 24 h
Yan et al. (21)	Observational	62	58	61.34	38/24	32/26	IV t-PA	Stent retrieval	Determined by study protocol	A intra-arterial bolus (10 ug/kg) followed by continuous intravenous infusion of 0.15 μg/kg/min for 24 h
Guo qiang et al. (22)	RCT	40	40	58.2	24/16	22/18	IV t-PA	Stent retrieval	Determined by study protocol	A intra-arterial bolus (6 ug/kg) followed by continuous intravenous infusion of 0.1 μg/kg/min for 24 h

*T, tirofiban group; N, non-tirofiban group; MT, mechanical thrombectomy; EVT, endovascular treatment; IVT, intravenous thrombolysis; na, not available; IVt-PA, intravenous tissue plasminogen activator*.

### Quality Evaluation of the Included Literature

A total of seven (16–22) studies were included, of which one (22) study was an RCT and six studies (16–21) were observational studies, and the quality of randomized controlled trials was evaluated using RoB 2.0 tool (Supplementary Figure 1), and observational studies were evaluated using NOS quality (Supplementary Table 1); in conclusion, the quality scores of the included literature were high, describing the selection of study populations and comparability between groups.

### Efficacy of Tirofiban

**Figure 2:**
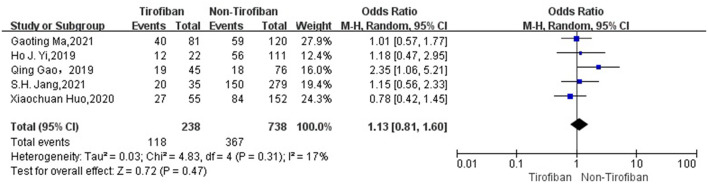
Forest plot and meta-analysis of 3-month mRS 0–2 score. mRS, modified Rankin Scale.

**Figure 3:**
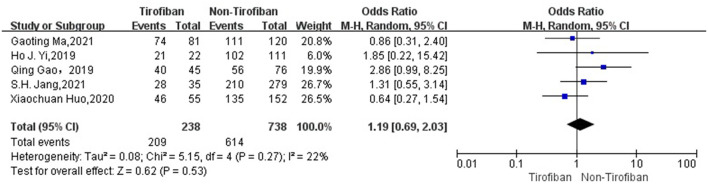
Forest plot and meta-analysis of postoperative recanalization rate.

In a total of 5 (16–20) studies included in the evaluation of favorable functional outcome at 3 months, 238 in the tirofiban group with 118(49.6%) MRS 0–2 scores and 738 in the non-tirofiban group with 367(49.7%) MRS 0–2 scores, with low heterogeneity (*I*^2^ = 17%, *p* = 0.31).The difference was not statistically significant in the 3-month MRS 0–2 scores between patients in the tirofiban and non-tirofiban groups (OR = 1.13, 95% Cl [0.81, 1.60], *p* = 0.47; shown in Figure 2). In terms of successful recanalization, a total of five (16–20) studies were included, with a recanalization rate of 87.8% (209/238) in the tirofiban group and 83.2% (614/738) in the non-tirofiban group, with no statistically significant difference (OR = 1.19, 95% Cl [0.69, 2.03], *p* = 0.53, *I*^2^ = 22%; shown in Figure 3).

### Safety of Tirofiban

**Figure 4:**
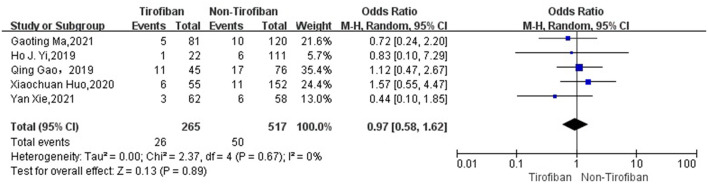
Forest plot and meta-analysis of the incident of sICH. sICH, symptomatic intracerebral hemorrhage.

**Figure 5:**
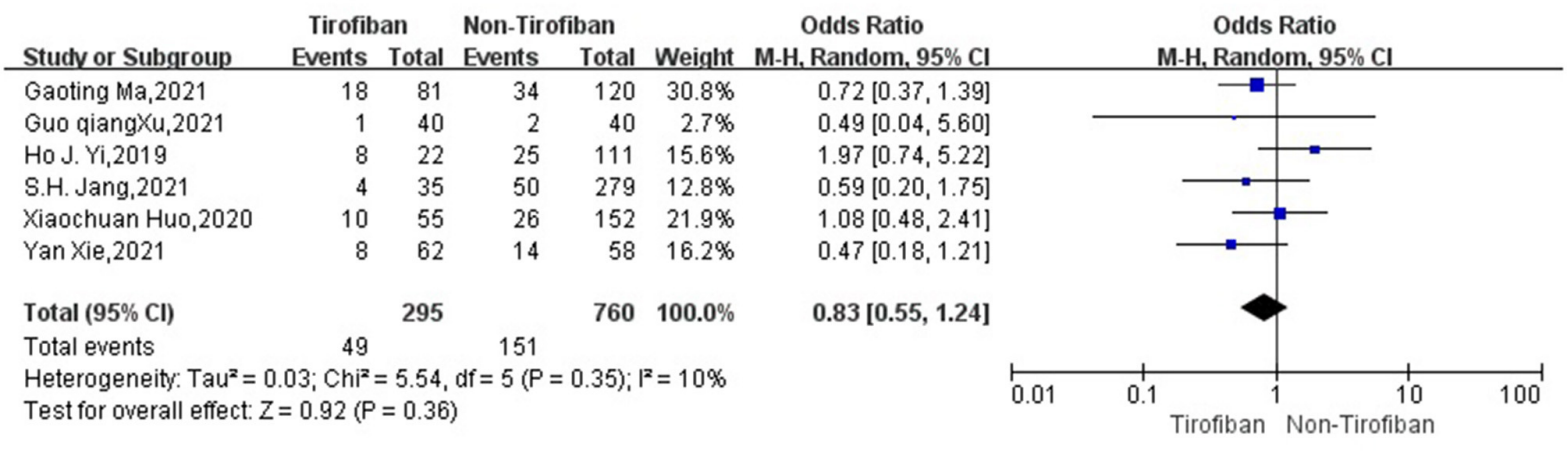
Forest plot and meta-analysis of the incident of ICH. ICH, intracerebral hemorrhage.

sICH data were available for 782 patients with low heterogeneity from 5 studies (16, 17, 19–21) (*p* = 0.67, *I*^2^ = 0%). sICH incidence was 9.8% (26/265) in the tirofiban group and 9.7% (50/517) in the non-tirofiban group. The difference was not statistically significant (OR = 0.97, 95% Cl [0.58, 1.62], *p* = 0.89; shown in Figure 4) in the tirofiban and non-tirofiban groups. A pooled analysis of six studies (16–21) evaluating ICH showed no statistically significant difference in the incidence of ICH between the tirofiban and non-tirofiban groups (OR = 0.83, 95% Cl [0.55, 1.24], *p* = 0.36; shown in Figure 5), with heterogeneity (*I*^2^ = 10%, *p* = 0.35).

**Figure 6:**
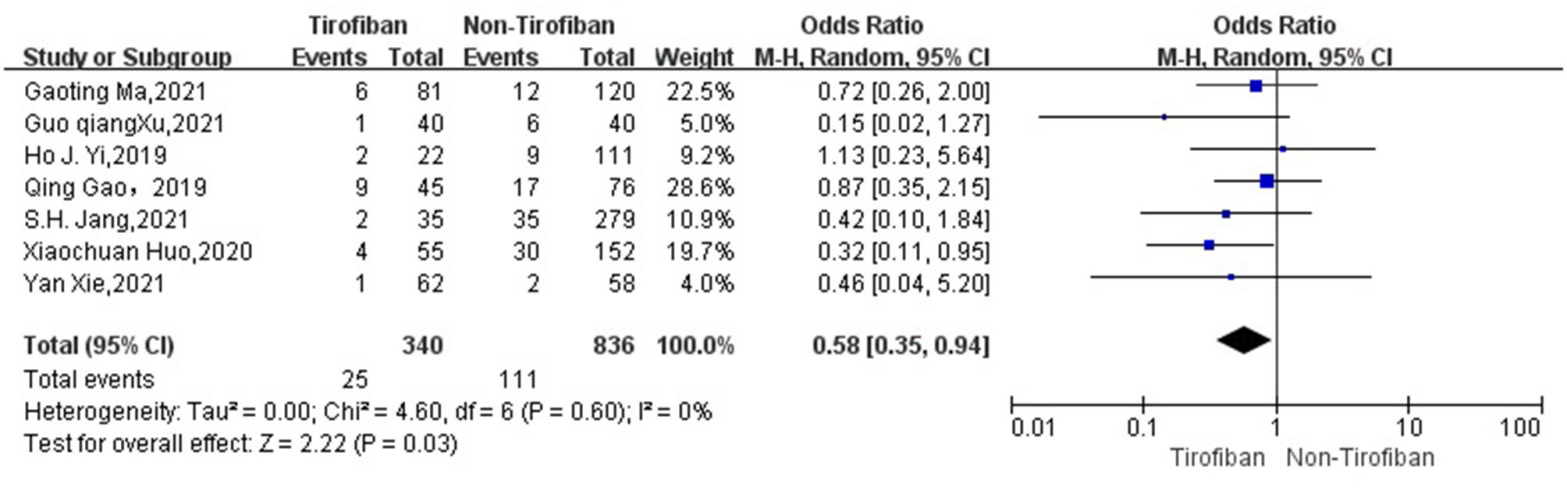
Forest plot and meta-analysis of mortality at 3 months.

**Figure 7:**
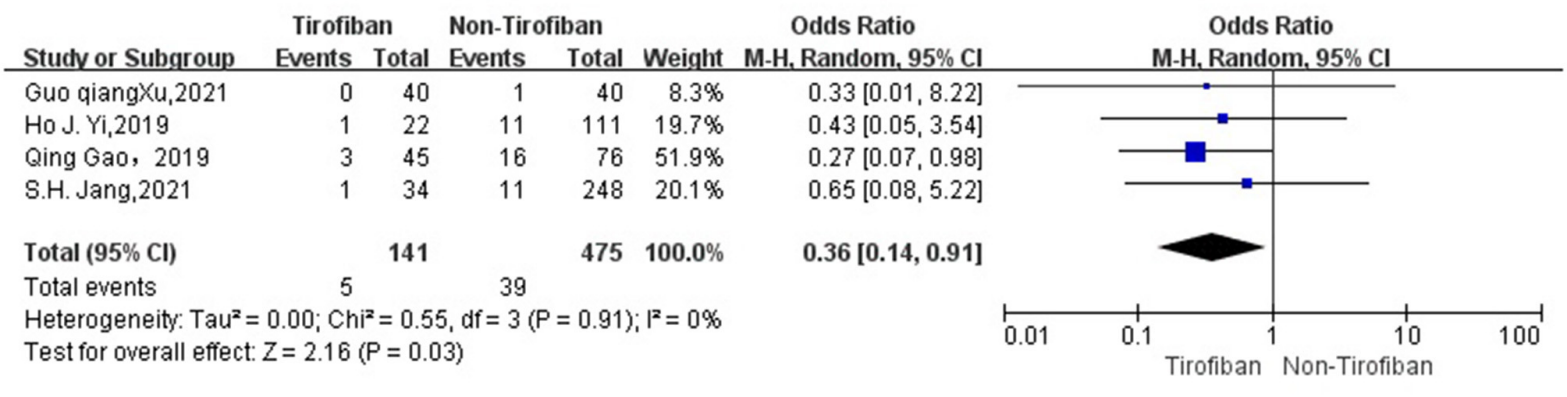
Forest plot and meta-analysis of Postprocedural re-occlusion.

The seven studies (16–22) reported mortality at 3 months in 1176 patients with AIS with low heterogeneity (*I*^2^ = 0%, *p* = 0.6), with 25 deaths in 340 patients in the tirofiban group compared to 111 deaths in 836 patients in the non-tirofiban group, with a lower mortality rate at 3 months in the tirofiban group than in the non-tirofiban group, with a statistically significant difference (OR = 0.58, 95% Cl [0.35, 0.94], *p* = 0.03; shown in Figure 6).For the assessment of postoperative re-occlusion in patients, meta-analysis included 4 studies with a total of 616 patients, with a postoperative re-occlusion rate of 3.5% (5/141) in the tirofiban group and 8.2% (39/475) in the non-tirofiban group, with a statistically significant difference between them (OR = 0.36, 95% Cl [0.14, 0.91], *p* = 0.03; shown in Figure 7), with heterogeneity (*I*^2^ = 0%, *p* = 0.91).

### Sensitivity Analysis and Publication Bias

In this meta -analysis, the results of the sensitivity analysis for efficacy and safety were consistent with the results of the combined analysis (Supplementary Figures 2–7); we used the Begg's and Egger's tests to assess the effect of publication bias, and the funnel plots were both symmetrical, with no significant evidence of publication bias (Supplementary Figures 8–13).

## Discussion

The efficacy and safety of antiplatelet agents in patients with acute ischemic stroke bridged by intravenous thrombolysis to mechanical thrombectomy remains unclear in clinical practice. A total of 7 papers involving 1,176 patients were included in this meta-analysis, and a comprehensive analysis showed no significant differences in the efficacy of tirofiban in terms of recanalization rates and favorable functional outcome at 3 months (MRS score 0–2) in patients with bridged mechanical thrombectomy after intravenous thrombolysis. In terms of safety, this meta-analysis showed that the use of tirofiban during IVT bridging MT did not increase the risk of sICH or ICH and reduced the rate of postoperative re-occlusion and mortality within 3 months in patients undergoing IVT bridging MT in acute ischemic stroke.

Tirofiban, a glycoprotein IIb/IIIa receptor antagonist, is widely used in percutaneous coronary interventions in patients with acute myocardial infarction, mainly to prevent platelet aggregation and thrombosis by reversibly blocking fibrinogen-binding receptors and modulating the final pathway of platelet aggregation (10). Tirofiban is limited in the treatment of cerebrovascular disease due to the risk of hemorrhagic transformation. Although in some clinical trials, tirofiban was used by intravenous or arterial administration for the acute treatment of endothelial damage at the stenosis site and *in situ* atherosclerotic stenosis during acute ischemic stroke MT (12, 23), the results remain controversial. There are even fewer studies on the safety of perioperative use of tirofiban in patients with IVT bridging MT. Kellert et al. (23) showed that treatment with tirofiban increased the risk of fatal cerebral hemorrhage, regardless of whether MT received IVT preoperatively. Risk and poor prognosis, which may be related to the higher intraoperative dose of tirofiban administration or the higher proportion of patients with cardiogenic stroke in their subject population. A meta-analysis by Fu et al. (24) showed that the perioperative use of tirofiban in EVT did not increase the risk of SICH and reduced mortality in patients, and a subgroup analysis showed that the preoperative use of tirofiban improved the favorable functional outcome of patients after surgery. However, a meta-analysis by Gong et al. (25) showed that EVT perioperative use of tirofiban increased the risk of ICH and did not improve patients' favorable functional outcome at 3 months, and a subgroup analysis showed that increased risk of ICH was strongly associated with intra-arterial injection of tirofiban. This meta-analysis of the safety and efficacy of tirofiban in patients undergoing IVT bridging MT found that the use of tirofiban in the perioperative period of IVT bridging MT did not increase the risk of sICH, ICH, which is the same as the results of Fu. In terms of improved functional outcome, our results are identical to those of Gong's study, and the use of tirofiban in IVT bridging MT did not improve the favorable functional outcome of patients at 3 months. However, our study found that the use of tirofiban in IVT bridging MT reduced the risk of postoperative vascular re-occlusion in patients, which is of great significance to us.

In a study of predictors of early arterial reocclusion after recanalization of IVT ischemic stroke, it was shown that r-tPA itself can mediate platelet activation and platelet inflammatory response through stimulation of fibrin production, promote platelet aggregation, and lead to secondary thrombosis, distal microcirculatory impairment, and even large vessel reocclusion (26). Antiplatelet aggregation therapy after IVT is theoretically feasible. However, a randomized controlled trial by Zinkstok et al. (27) in 2012 suggested that early oral antiplatelet agents (aspirin) after IVT in patients with AIS did not improve outcome at 3 months, but rather increased the risk of SICH in the AHA/ASA guidelines, antiplatelet agents are not recommended within 24 h after IVT because of concerns about bleeding complications increase (14). However, compared to oral antiplatelet agents such as aspirin, tirofiban, when administered intravenously, has a rapid onset of action, short time to peak, short half-life of action, reversible antiplatelet effects, and rapid recovery of platelet function about 4 h after discontinuation, greatly reducing the risk of bleeding 2019. Wu et al. (28) have demonstrated that early use of tirofiban in patients with neurological deterioration within 24 h after IVT Low-dose tirofiban does not increase the risk of symptomatic intracranial hemorrhage, cerebral hemorrhage, and mortality, which is in line with the conclusions reached in this meta-analysis. In other words, the dose of tirofiban administered in the original literature included in this meta-analysis (0.25–1 mg by initial intra-arterial injection, followed by continuous intravenous pumping at 0.1 ug/kg/min for ~12–24 h) is safe. And, in this study, we also found that the use of tirofiban during IVT bridging MT substantially reduced the risk of postoperative re-occlusion, which further supports the feasibility of using tirofiban as an antiplatelet agent during IVT bridging MT. However, in 2020 Yang et al. (29) showed through a study that the efficacy of tirofiban depends on its route of administration, and that intravenous tirofiban was associated with increased recanalization rates and improved 3-month favorable functional outcome after EVT in patients with acute ischemic stroke. In contrast, intra-arterial administration of tirofiban was associated with an increased incidence of symptomatic cerebral hemorrhage and a poor 3-month functional outcome. Large-scale clinical trials are still needed to investigate the specific safe dosing and administration of tirofiban.

The present meta-analysis showed that tirofiban reduced mortality within 3 months in patients with IVT bridging to MT, which is consistent with previous findings published by Huo et al. (17) and a meta-analysis by Chen et al. (30), who also found that in acute ischemic stroke combined with large vessel occlusion, intra-arterial adjuvant drugs (IAM) such as urokinase, tissue-type fibrinogen activator (TPA) or glycoprotein IIb/IIIa inhibitors combined with MT resulted in a better functional outcome and lower mortality. When a large artery is recanalized after occlusion no-reflow phenomenon occurs due to persistent distal emboli and microcirculatory occlusion that does not produce effective tissue perfusion, but the combination of MT with agents such as tirofiban can prevent this phenomenon, thereby reducing tissue ischemia and eventual infarct volume, resulting in better functional outcomes and lower mortality in patients. However, this meta-analysis found no statistical difference in functional improvement with tirofiban, and in some studies (16, 18–21) did not find that tirofiban reduced mortality within 3 months in patients with IVT bridging to MT, which we speculate may be related to the small sample size of the study, so a large clinical trial is still needed to study and verify it. This meta-analysis did not perform a subgroup analysis of ischemic stroke etiology because most of the included patients were patients with atherosclerotic large vessel occlusions. It has been found (31, 32) that tirofiban appears to have a good functional outcome with lower mortality in patients with large atherosclerotic stroke, but patients with cardiogenic embolic stroke tend to have a poor prognosis, and the reasons for this outcome are mostly attributed to the thrombotic component. Cardiogenic embolism is mainly red thrombus rich in red blood cells, while embolism induced by large artery atherosclerotic type is mainly white thrombus composed of platelets. Tirofiban, as a platelet antagonist, inhibits platelet aggregation and benefits patients with large artery atherosclerotic stroke by maintaining reperfusion. In addition, in patients with intracranial atherosclerosis-related stroke, tirofiban stabilizes inflammatory stenotic lesions and maintains blood flow, which can help prevent some ischemic events caused by inflammation and platelet aggregation (24). Therefore, patients with atherosclerotic occlusions of large arteries may benefit more from tirofiban.

## Limitations

In interpreting the results, a number of limitations should be highlighted. First, most of the included studies were observational studies and selection bias was inevitable, and this selection bias may have artificially reduced the effectiveness of tirofiban in improving prognosis. Second, not all of the studies had the data needed to assess the safety and efficacy of tirofiban. Third, the overall sample size of this study was small, which may have influenced the results. Fourth, the majority of the population included in this study was from Asia and the results may be applicable to Asian patients but not to Western patients.

## Conclusion

The use of tirofiban during IVT bridging to mechanical thrombectomy for AIS does not increase the risk of sICH and ICH in patients and reduces the risk of postoperative re-occlusion and mortality in patients within 3 months. However, more large-sample, multicenter, prospective randomized controlled trials are needed for further confirmation.

## Data Availability Statement

The original contributions presented in the study are included in the article/Supplementary Material, further inquiries can be directed to the corresponding author.

## Author Contributions

WL participated in the design of the study, collected and analyzed the data, and drafted and revised the manuscript. GL, ZX, YZha, BL, and YZho analyzed the data, interpreted the results, and performed the statistical analysis. EC designed the study, supervised the study inclusion and data extraction, and revised the manuscript. All authors contributed to the article and approved the submitted version.

## Conflict of Interest

The authors declare that the research was conducted in the absence of any commercial or financial relationships that could be construed as a potential conflict of interest.

## Publisher's Note

All claims expressed in this article are solely those of the authors and do not necessarily represent those of their affiliated organizations, or those of the publisher, the editors and the reviewers. Any product that may be evaluated in this article, or claim that may be made by its manufacturer, is not guaranteed or endorsed by the publisher.
