# Supplementary material for: Safety and Efficacy of Tirofiban During Intravenous Thrombolysis Bridging to Mechanical Thrombectomy for Acute Ischemic Stroke Patients: A Meta-Analysis
Source: Front Neurol. 2022 Apr 29;13:851910. doi: 10.3389/fneur.2022.851910 (PMC9099208; doi:10.3389/fneur.2022.851910)
Supplement: Supplementary file 1 [file Table_1.docx]

**Supplementary Figures**

Supplementary Figure 2. The results of a sensitivity analysis for 3-month MRS 0–2 score. MRS, modified Rankin Scale.

Supplementary Figure 3. The results of a sensitivity analysis for postoperative recanalization rate.

Supplementary Figure 4. The results of a sensitivity analysis for the incident of sICH. sICH, symptomatic intracerebral hemorrhage.

Supplementary Figure 5. The results of a sensitivity analysis for the incident of ICH. ICH,intracerebral hemorrhage.

Supplementary Figure 6. The results of a sensitivity analysis for mortality at 3 months.

Supplementary Figure 7. The results of a sensitivity analysis for Post-procedural re-occlusion.

Supplementary Figure 8. A funnel plot for assessing publication bias in regards to 3-month mRS 0–2 score. mRS, modified Rankin Scale.

Supplementary Figure 9. A funnel plot for assessing publication bias in regards to postoperative recanalization rate.

Supplementary Figure 10. A funnel plot for assessing publication bias in regards to the incident of sICH. sICH, symptomatic intracerebral hemorrhage.

Supplementary Figure 11. A funnel plot for assessing publication bias in regards to the incident of ICH. ICH, intracerebral hemorrhage.

Supplementary Figure 12. A funnel plot for assessing publication bias in regards to

mortality at 3 months.

Supplementary Figure 13. A funnel plot for assessing publication bias in regards to Postprocedural re-occlusion.

**Search Type:**

(((((((((((((((((Ischemic Stroke[Title/Abstract])) OR (Ischemic Strokes[Title/Abstract])) OR (Stroke, Ischemic[Title/Abstract])) OR (Ischaemic Stroke[Title/Abstract])) OR (Ischaemic Strokes[Title/Abstract])) OR (Stroke, Ischaemic[Title/Abstract])) OR (large vessel occlusion [Title/Abstract])) OR (large artery occlusions[Title/Abstract])) OR (Wake-up Stroke[Title/Abstract])) OR (Stroke, Wake-up[Title/Abstract])) OR (Wake up Stroke[Title/Abstract])) OR (Wake-up Strokes[Title/Abstract])) OR (Acute Ischemic Stroke[Title/Abstract])) OR (Acute Ischemic Strokes[Title/Abstract])) OR (Ischemic Stroke, Acute[Title/Abstract])) OR (Stroke, Acute Ischemic[Title/Abstract]))AND ((((((((((((((Tirofiban[Title/Abstract])) OR (GP IIb/IIIa receptor antagonist[Title/Abstract])) OR ( Aggrastat[Title/Abstract])) OR (Agrastat[Title/Abstract])) OR (MK 383[Title/Abstract])) OR (MK-383[Title/Abstract])) OR (L 700462[Title/Abstract])) OR (L-700462[Title/Abstract])) OR (L-700,462[Title/Abstract])) OR (L 700,462[Title/Abstract])) OR (L700,462[Title/Abstract])) OR (Tirofiban Hydrochloride[Title/Abstract])) OR (Tirofiban Hydrochloride Monohydrate[Title/Abstract])))AND(((((mechanical thrombectomy[Title/Abstract])) OR (intravenous thrombolysis[Title/Abstract])) OR (bridging therapy[Title/Abstract]))

Supplementary Figure 1: Inclusion in randomized controlled trials using Risk-of-bias tool (RoB 2.0)


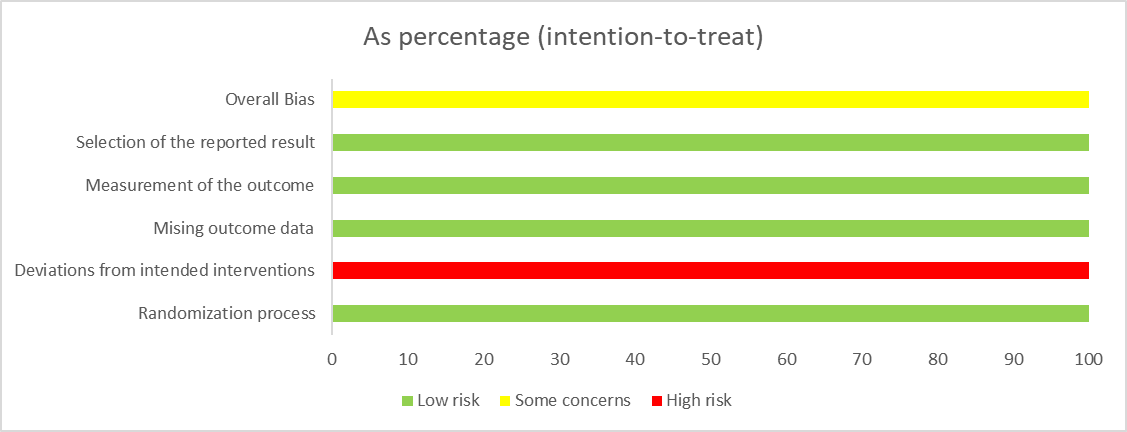


Supplementary Table1:Inclusion of observational studies literature NOS evaluation scales

| Study | Selection | | | | Comparability  Control for important factor | Exposure | | | Scores |
| --- | --- | --- | --- | --- | --- | --- | --- | --- | --- |
|  | Adequate definition of cases | Representati-veness of the cases | Selection of controls | Definition of controls |  | Ascertainment of exposure | Same method Of ascertainment for Cases And Controls | Non-responserate |  |
| Ho J. Yi et al.[16] | 1 | 1 | 0 | 1 | 2 | 1 | 1 | 1 | 8 |
| Xiaochuan Huo etal [17] | 1 | 1 | 0 | 1 | 2 | 1 | 1 | 1 | 8 |
| S.H. Jang et al.[18] | 1 | 1 | 0 | 1 | 2 | 1 | 1 | 1 | 8 |
| Gaoting Ma et al.[19] | 1 | 1 | 0 | 1 | 2 | 1 | 1 | 1 | 8 |
| Qing Gao et al[20] | 1 | 1 | 0 | 1 | 1 | 1 | 1 | 1 | 7 |
| Yan XIE  et al[21] | 1 | 1 | 0 | 1 | 1 | 1 | 1 | 1 | 7 |
